# Correction

**DOI:** 10.1080/21505594.2026.2664971

**Published:** 2026-04-29

**Article title**: Crystal structure of the *Legionella pneumophila* effector SidL (Lpg0437) in complex with its metaeffector LegA11 (Lpg0436)

**Authors**: Dominik A. Machtens, Carissa A. Hutchison, Ashley M. Stein, Jan Eberhage, Jonas M. Willerding, Susanne Eschenburg, Stephanie R. Shames, and Thomas F. Reubold

**Journal**: *VIRULENCE*

**DOI**: http://dx.doi.org/10.1080/21505594.2026.2646775

When the article was published online, the value was inadvertently missed to include in Table 1. Which is now corrected and the article is re-published online.

**Table 1.** Data collection and refinement statistics.

	SidL/LegA11 native	SidL/LegA11 SeMet
**Data collection**		
Wavelength (Å)	0.978565	0.97950
Space group	P 21 21 21	P 21 21 21
Unit cell *a*, *b*, *c* (Å)	68.53, 93.38, 133.67	69.65, 97.38, 130.30
Resolution range (Å)	47.85 - 2.4 (2.486 - 2.4)^a^	78.003 – 3.3 (3.4 – 3.3)
CC_1/2_	0.999 (0.665)	0.999 (0.892)
R_meas_^b^	0.1425 (1.411)	0.304 (2.02)
Total reflections	288,234 (37736)	696,282 (62675)
Unique reflections	41,606 (5384)	25,754 (2217)
Completeness (%)	99.70 (100.00)	100.00 (100.00)
Redundancy	6.9 (7.0)	27.0 (28.3)
<*I*/σ(*I)*>	23.42 (2.04)	16.13 (3.87)
**Structure refinement**		
Resolution range (Å)	47.85 - 2.4 (2.47 – 2.40)	
R_work_^c^	0.2244 (0.2995)	
R_free_^d^	0.2672 (0.3812)	
No. of atoms	5658	
No. of protein atoms	5472	
No. of water molecules	186	
B-factors protein/water	66.71/59.32	
R.m.s.d. Bond lengths (Å)	0.003	
R.m.s.d. Bond angles (°)	0.56^e^^d^	
Protein Data Bank entry	9SXC	

^a^The values in parentheses refer to statistics in the highest bin.

^b^R_meas_ = Σ_hkl_ (n_hkl_/n_hkl_ − 1)^1/2^ Σ_i_|<I_hkl_ > −I_hkl,i_|/Σ_hkl_ Σ_i_ I_hkl,i_

^c^R_work_ = Σ_hkl_|F_o_(hkl)-F_c_(hkl)|/Σ_hkl_F_o_(hkl), where F_o_ and F_c_ are the observed and calculated structure-factor amplitudes, respectively.

^d^R_free_ was calculated with 5% of the data excluded from the refinement.

^e^R.m.s.d. = root mean square deviation.

