# Supplementary material for: Crystal structure of the Legionella pneumophila effector SidL (Lpg0437) in complex with its metaeffector LegA11 (Lpg0436)
Source: Virulence. 2026 Mar 17;17(1):2646775. doi: 10.1080/21505594.2026.2646775 (PMC13007446; doi:10.1080/21505594.2026.2646775)
Supplement: Supplemental Information_v1.docx [file KVIR_A_2646775_SM8275.docx]

**Supplemental Information**

**Suppl. Table 1.** Oligonucleotide primers used in this study

| Name | Sequence (5′🡪3′) |
| --- | --- |
| LegA11_KO1-F | ATT**GTCGAC**GGGATCACCAATCGATGGACG |
| LegA11_KO1-R | ATT**GCGGCCGC**ACACCTATTTTCTATCGCCATAG |
| LegA11_KO2-F | ATT**GCGGCCGC**ATAAATTTTTTATCCTATCTATTAA |
| LegA11_KO2-R | ATT**GAGCTC**GATCAATCTATCTAAATCGGC |
| SidL_KO1-F | ATT**GTCGAC**CCGGTCATAAAGAAAAGAC |
| SidL_KO1-R | ATT**GCGGCCGC**ACATATAACCCTCTACCTCTTAG |
| SidL_KO2-F | ATT**GCGGCCGC**ATAAAGGATAATTTGGGTTCC |
| SidL_KO2-R | ATT**GAGCTC**CCCTAAAAGTGACTATTCAC |
| LegA11JBBamHI-F | ATT**GGATCC**TTGTGATTAAAATGGGTAGAAG |
| Lega11BamHI-F | ATT**GGATCC**GTGATTAAAATGGGTAGAAG |
| LegA11SalI-R | ATT**GTCGAC**TTAAAGTGCGTTTTTAGGGG |
| LegA11Not1-R | ATT**GCGGCCGC**TAAAGTGCGTTTTTAGGGG |
| SidLJBBamHI-F | ATT**GGATCC**TTATGCAAAACTTAGATGAGATTC |
| SidLBamH1-F | ATT**GGATCC**ATGCAAAACTTAGATGAGATTC |
| SidLSalI-R | ATT**GTCGAC**TTAGCACCCATAAACAGTTC |
| SidL_1s_BamHI | ACA**GGATCC**ATGCAAAACTTAGATGAGATTCTAAAGAAACTG |
| SidL_23s_BamHI | ACA**GGATCC**ATGACTGAGAAGCTCAGTGTTTTATCAAAGAAA |
| SidL_76s_BamHI | GCAC**GGATCC**AATATACGTTTTTCTCGATTTAAGCAG |
| SidL_640as_SalI | CGC**GTCGAC**TTATGAATTTGATTTGAGTGCTTCC |
| SidL_645as_SalI | GCAC**GTCGAC**TCATTTCTTACTGAAGAATGAATTTGATTTG |
| SidL_666as_SalI | CGC**GTCGAC**TTAGCACCCATAAACAGTTCCATCTTTTGGC |
| LegA11_1s_BamHI | GCAC**GGATCC**ATGATTAAAATGGGTAGAAGTGAAATG |
| LegA11_269as_SalI | GCAC**GTCGAC**TCAAAGTGCGTTTTTAGGGGTATCTATGCC |

**Bold** text denotes restriction endonuclease cleavage sites


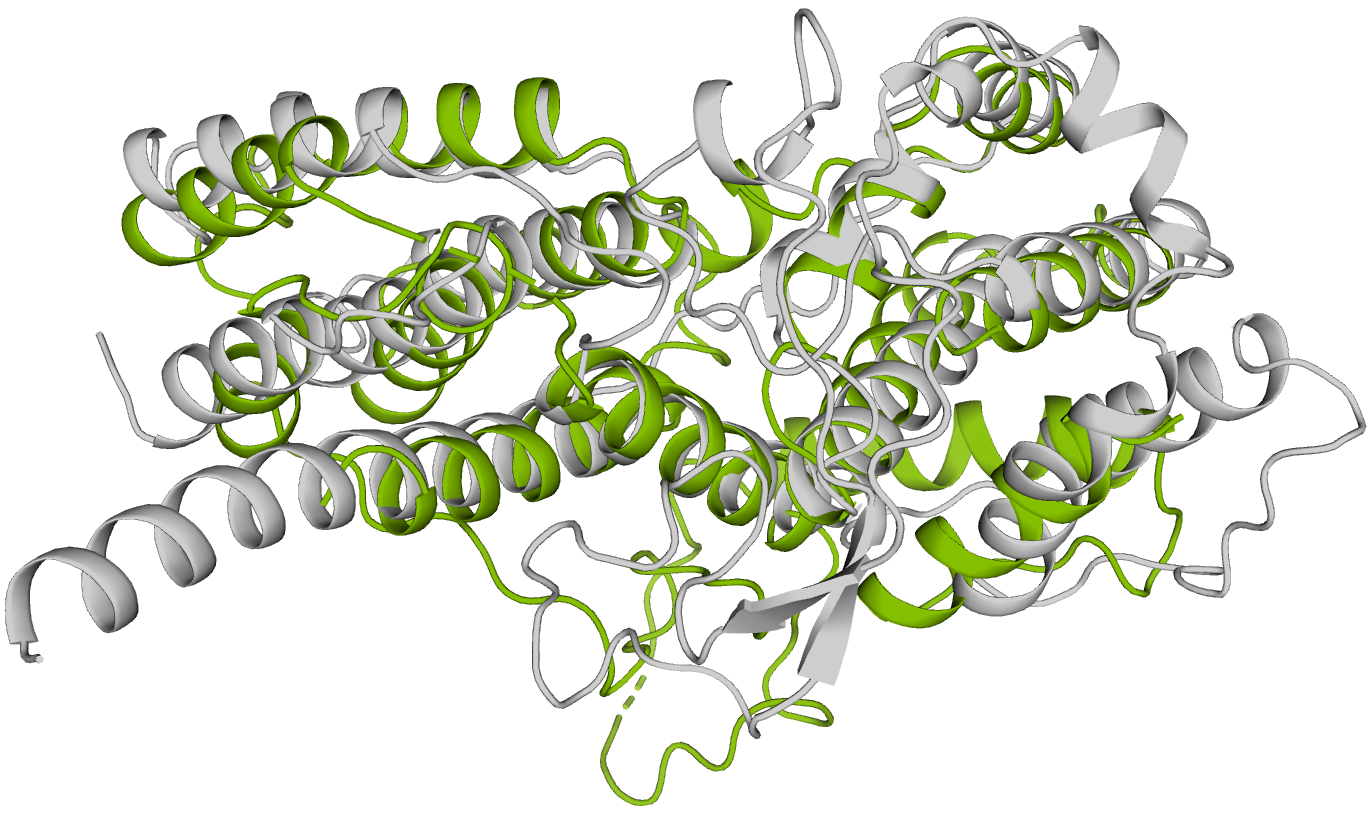


**Suppl. Fig. 1. Superposition of SidL and LnaB.** Cartoon representation of the three-dimensional structure of SidL_C_ superposed to a crystal structure of LnaB of *L. pneumophila* (PDB ID 8JO3). SidL is shown in green, LnaB is shown in gray.


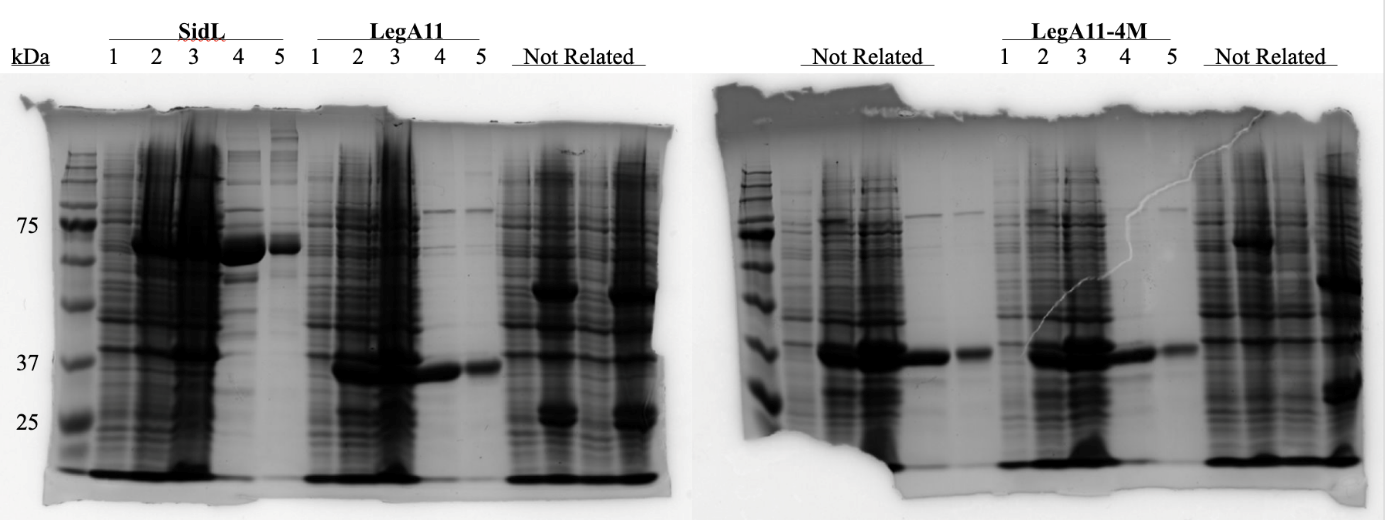


**Suppl. Fig 2. Purification of recombinant SidL and LegA11 for in vitro translation assays. P**urification of recombinant SidL and LegAll variants was visualized via SDS-Page and Coomassie Brilliant Blue staining. Proteins for each set of lanes denoted above. Lanes numbered as follows: 1) Uninduced 2) Induced 3) Sonicated 4) Purified protein 5) Dialysed purified protein.


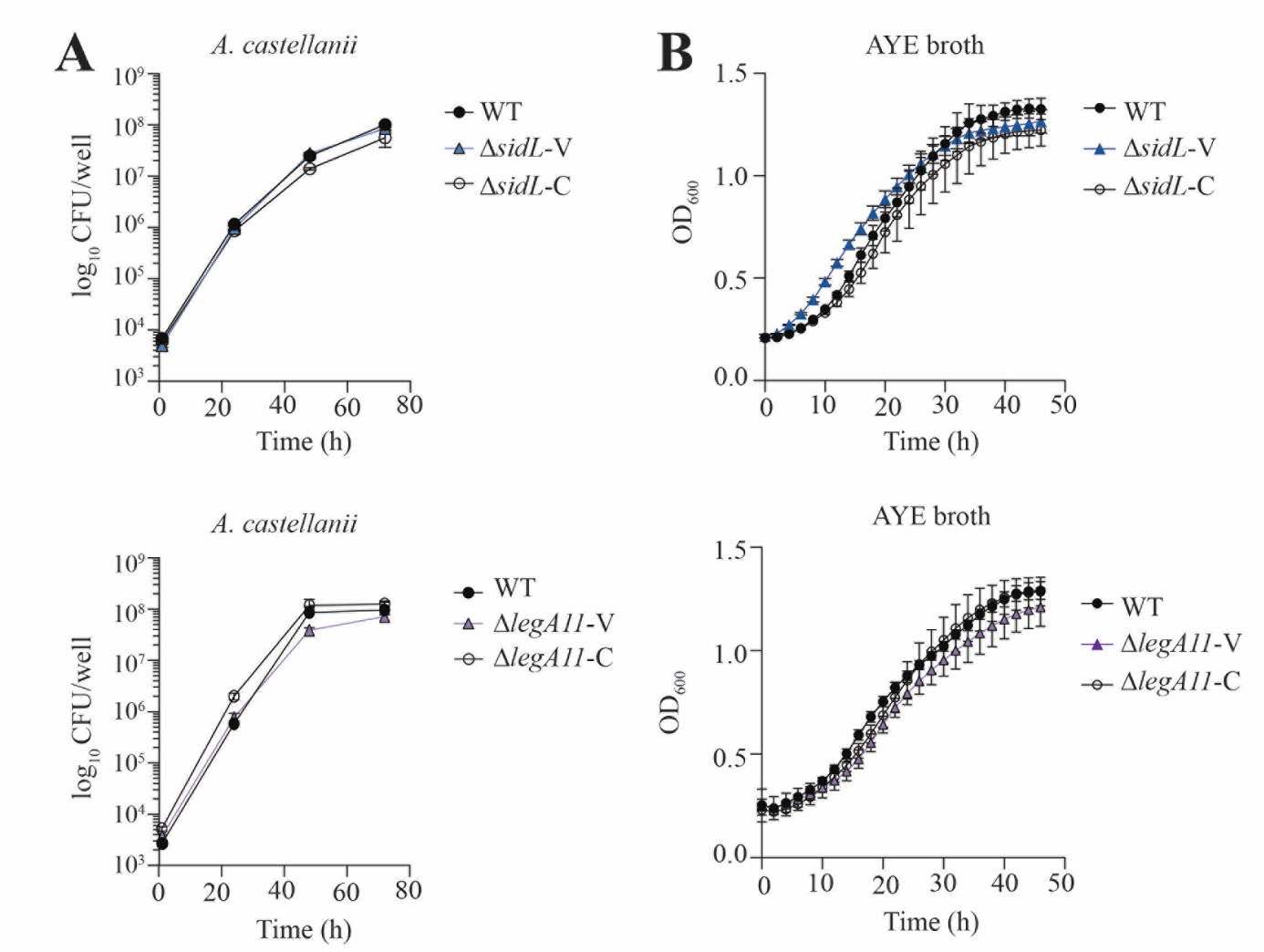


**Suppl. Fig. 3. Neither SidL nor LegA11 impact *L. pneumophila* intracellular replication or growth *in vitro*. (A)** Colony forming units (CFU) recovered from *Acanthamoeba castellanii* infected with the indicated *L. pneumophila* strains (MOI of 0.1) for the indicated times. **(B)** Optical density (OD) 600 nm values of *L. pneumophila* grown in AYE broth. Data were collected every 2h for up to 46h and shown as mean ± standard deviation (s.d.) of six individual wells (N=6). Representative of two independent experiments. Data shown are mean ± s.d. of three independent wells (N=3) and representative of three independent experiments. V denotes empty plasmid vector, and C denotes plasmid-based genetic complementation construct.


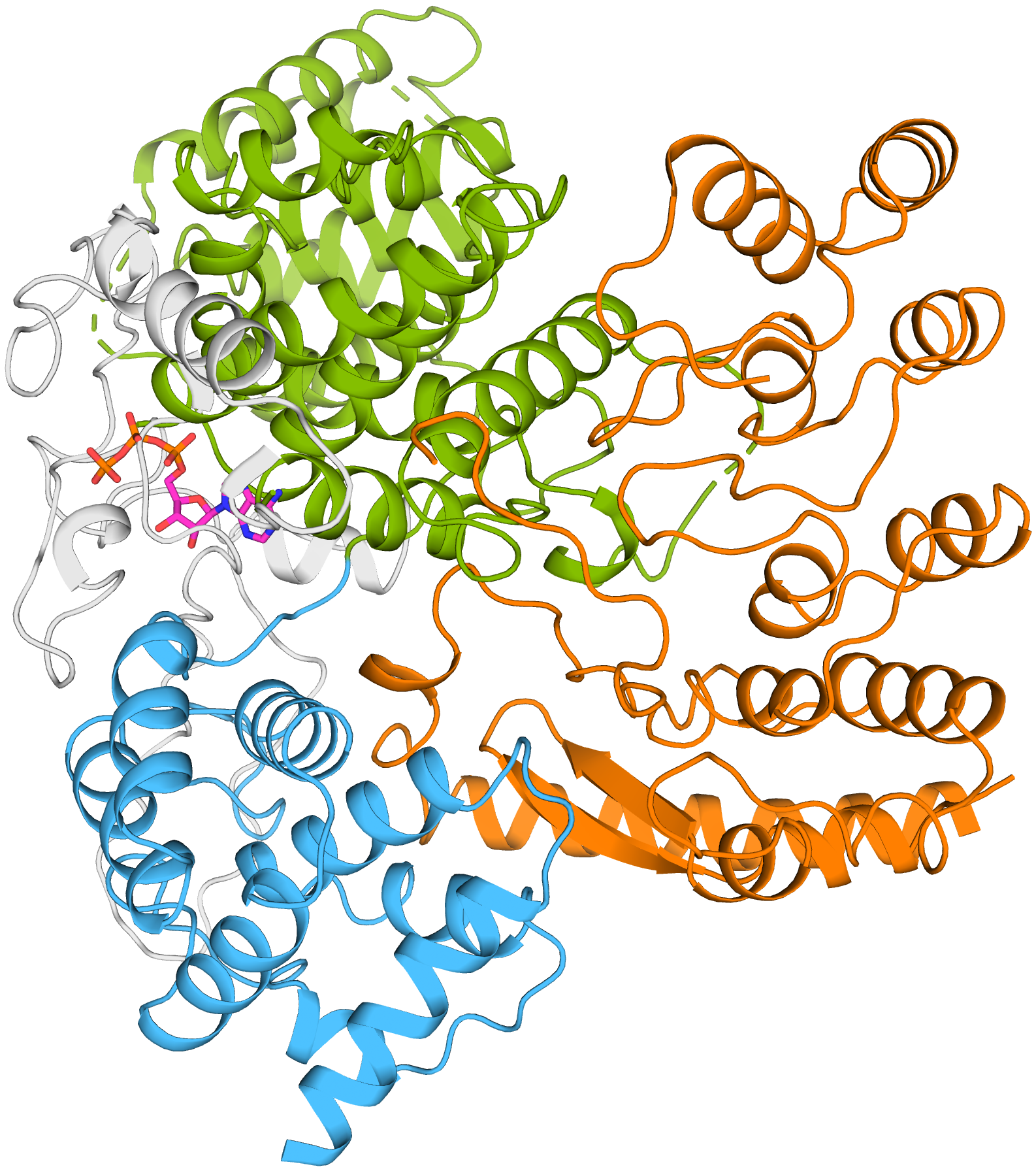


**Suppl. Fig. 4. Spatial relationship between the binding sites of LegA11 and ATP in SidL.** Cartoon representation of the crystal structure of the SidL/LegA11 complex. The color code corresponds to that in Fig. 2. The ATP molecule from an AF3 prediction of SidL complexed with ATP is shown as stick model. The coloring of the ATP is as follows: carbon – magenta; nitrogen – blue; oxygen – red; phosphorus – orange.


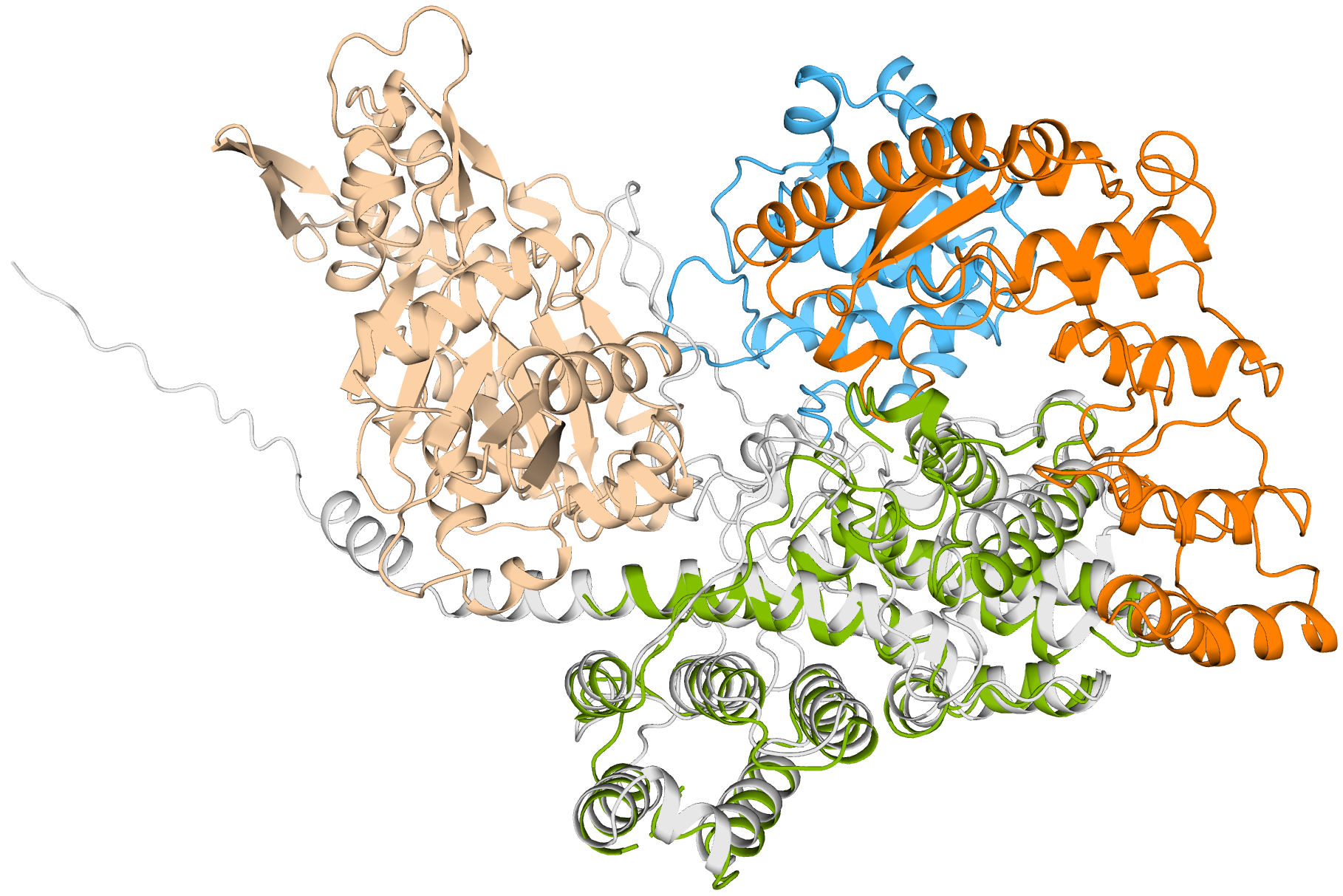


**Suppl. Fig. 5. Hypothetical ternary complex of SidL, LegA11, and actin.** SidL from the crystal structure was superposed with SidL_C_ from an AF3 prediction of SidL and actin. Components of the crystal structure are colored as in Fig. 2, SidL_C_ from the AF3 prediction is shown in gray, actin is shown in beige.


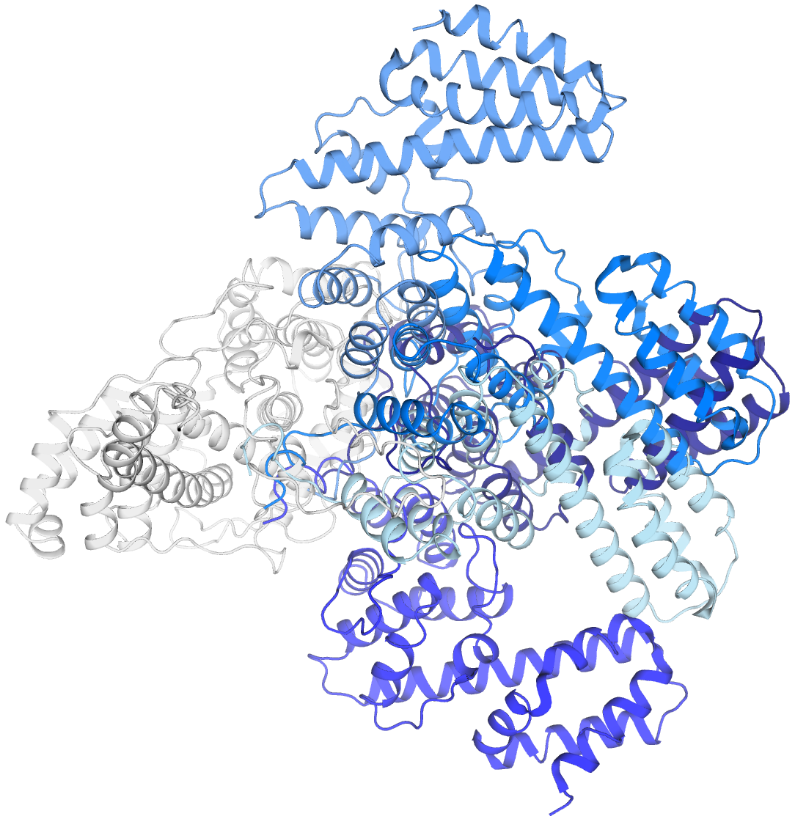


**Suppl. Fig. 6. Conformational flexibility of SidL_N_ in AF3 predictions of complexes between SidL and actin.** Cartoon representation of a superposition of the SidL molecules of five AF3 predictions of a SidL/actin complex. The models are superposed via SidL_C_. SidL_N_ of the different models are shown in different shades of blue.
